# Preliminary Investigation on the Physicochemical and Functional Properties of Commercial *Salmorejo* Found in Spanish Supermarkets

**DOI:** 10.3390/foods10051146

**Published:** 2021-05-20

**Authors:** Montserrat Vioque, Silvia de la Cruz-Ares, Rafael Gómez

**Affiliations:** Department of Food Science and Technology, Campus de Rabanales, Darwin Building, University of Córdoba, 14014 Cordoba, Spain; bt1viamm@uco.es (M.V.); scruz@uco.es (S.d.l.C.-A.)

**Keywords:** Mediterranean diet, DPPH• radical scavenging capacity, polyphenols, lycopene

## Abstract

*Salmorejo* is a traditional Spanish food made of raw tomatoes, bread, garlic, and virgin olive oil. The food industry aims to satisfy consumer demand for ready-to-eat *salmorejo* while trying to maintain characteristics of the homemade product. In this work, we have assessed physical-chemical and color parameters, radical scavenging activity, and total polyphenol, lycopene, and β-carotene contents of raw and pasteurized commercial *salmorejo*, as well as homemade (raw) *salmorejo* samples. Our results showed that heat treatment had a significant influence on color parameters of *salmorejo*, with pasteurized samples being less red and exhibiting an increase in the degree of browning. Pasteurized samples also showed the highest radical scavenging activity when expressing the results per dry weight. However, when water content was considered, radical scavenging activity was superior in homemade samples when analyzing non-polar molecules extracted with acetone. Results were similar for polyphenol content. It was also observed that heat treatment affected lycopene but not β-carotene content. When acquiring commercial *salmorejo* at the supermarket, consumers have the option to choose between already prepared pasteurized or raw *salmorejo*. According to the results obtained in this work, physicochemical and functional properties of commercial raw *salmorejo* were comparable to a larger extent than pasteurized *salmorejo* compared to those exhibited by homemade *salmorejo* samples.

## 1. Introduction

According to the WHO Global Health Estimates report for the year 2016, the leading causes of death in the world were ischemic heart disease (16.6%) and stroke (10.2%). Alzheimer’s disease and other dementias (3.5%), diabetes mellitus (2.8%), and colon and rectum cancers (1.4%) were also among the top 20 causes of death among population [1].

Diet and lifestyle are acknowledged to play a significant role in the prevention of chronic diseases. Adherence to a diet rich in fruits and vegetables contributes to reduce the incidence of conditions associated with cardiovascular diseases, such as obesity, metabolic syndrome, and type 2 diabetes mellitus, among other benefits [2,3].

Mediterranean diet (MD) is defined as a plant-based, antioxidant-rich dietary pattern found in the early 1960s in olive-growing countries of the Mediterranean basin. It is characterized by its richness in vegetables, fruits, legumes, seeds, and whole grain cereals, use of olive oil as the main source of fat, and limited consumption of confectionery and red or processed meats. Regarding nutrients provided by MD, mono- and polyunsaturated fatty acids, polyphenols and other bioactive compounds such as phytosterols are abundant, whereas saturated fats are scarce. Several epidemiological studies claim that adherence to MD is associated with longevity and improved quality of life due to its capacity to prevent age-related dysfunction and to reduce the risk of chronic diseases such as cardiovascular diseases, metabolic syndrome, age-related cognitive impairment, type 2 diabetes mellitus or cancer [4]. Those health benefits are partly credited to the remarkable antioxidant power of polyphenols and their implication in modulating oxidative pathways. Several studies have described an inverse correlation between total polyphenol, lignans, flavonols, or hydroxybenzoic acids intake and risk of cardiovascular-related events [5,6]. Moreover, a polyphenol rich diet might help to reduce systolic and diastolic blood pressure in elderly hypertensive populations [7]. Furthermore, oxidative stress, which is also involved in inflammatory diseases, cancer, and age-related cognitive decline and neurodegenerative diseases, might be counteracted by consuming antioxidant-rich foods [8].

*Salmorejo* is a traditional Andalusian food originated in Córdoba, that is often taken as appetizer or as starter. It consists of a puree prepared with tomatoes (*Solanum lycopersicum* L. (Solanaceae)), bread, garlic (*Allium sativum* L. (Amaryllidaceae)), virgin olive (*Olea europaea* L. (Oleaceae)) oil and salt, but can include additional vegetables (cucumber, (*Cucumis sativus* L. (Cucurbitaceae)), green pepper (*Capsicum annuum* L. (Solanaceae)), and onion (*Allium cepa* L. (Amaryllidaceae)), etc.) following local customs [9].

Owing to its ingredients, *salmorejo* is rich in vitamins, minerals, and antioxidant compounds. For instance, tomato contains a large variety of antioxidants such as polyphenols, carotenoids, and tocopherols. Lycopene might prevent in vivo oxidation of lipids, proteins and DNA thanks to its ability to trap free radicals. Water-soluble tomato extract possesses antiplatelet factors that inhibit platelet aggregation, therefore protecting the cardiovascular system [10,11]. On the other hand, several studies have reported that garlic supplementation reduces blood pressure, therefore affording cardioprotection in hypertensive populations. Down to its antioxidant compounds, garlic might also counteract oxidative stress, scavenging free radicals, and therefore inhibiting lipid peroxidation [12,13]. Phenolic compounds found in virgin olive oil (mainly oleuropein, tyrosol and hydroxytyrosol) improve cardiovascular risk factors, such as inflammation, oxidative stress, coagulation, endothelial function, etc. They have also shown anti-proliferative activities in several cancer studies carried out in vitro, and have exhibited positive effects in the management of non-alcoholic fatty liver disease [14,15,16]. Considering all the above, *salmorejo* intake would therefore contribute to maintaining a good health status, always in the context of an adequate lifestyle and a balanced diet.

Nowadays, consumers demand ready-to-eat food products that preserve organoleptic and nutritional properties, and food industry satisfies this request by offering goods preserved using different technologies. Regarding *salmorejo*, this product is usually presented as raw or pasteurized (sometimes combined with high pressure processing) and stored at cooling temperatures in supermarkets. Heat treatment destroys microorganisms and inactivates antinutritional factors while improving the flavor, digestibility, and palatability of foods. However, it affects the concentration and availability of bioactive compounds [17]. Currently, there are different opinions concerning the effect of heat treatments on the antioxidant activity of food products. Several studies declare that the thermal treatment of food improves the availability, absorption, and utilization of phenolic compounds because it improves polyphenol extraction and inactivates polyphenol oxidase. Nonetheless, others refer to losses in polyphenols and antioxidant capacity [18].

Taking these considerations into account, the objective of the present work has been to analyze physicochemical characteristics and functional properties of raw and pasteurized commercial *salmorejo* samples from different brands acquired in Spanish supermarkets to assess the extent to which commercial products differ from homemade ones.

## 2. Materials and Methods

### 2.1. Sample Selection

Commercial *salmorejo* samples were purchased from different local retailers in Córdoba (Spain) and kept at 4 °C until used. Sampling was performed based on product availability, and samples included 5 pasteurized (P) and 7 raw (R) products from different brands. Additionally, two homemade (H) *salmorejo* samples were prepared in-house following the recipe described by Moreno-Rojas et al. [9]. To that end, 1000 g *plum* tomato, 200 g bread, 5 g garlic, and 9 g of salt were blended at high speed at room temperature (RT) for 1 min. Then, 100 g extra virgin olive oil (EVOO) were slowly poured while blending at moderate speed until a homogenous mixture was obtained.

### 2.2. Reagents and Chemicals

All reagents used were of analytical grade and purchased at Panreac (Panreac Química S.L.U., Barcelona, Spain), unless stated otherwise.

### 2.3. pH and Water Activity Measurements

pH values were measured using a Hanna HI 2020 pH meter equipped with a digital electrode with an integrated temperature sensor (Hanna Instruments, Woonsocket, RI, USA), calibrated with buffer solutions of pH 4.04 and 7.01 (Hach, Loveland, CO, USA).

Water activity (a_w_) of *salmorejo* samples was determined using an AquaLab water activity meter (Decagon Devices, Model 4 TE, Pullman, WA, USA) following manufacturer’s instructions. Prior to performing the measurements, samples were homogenized, disposed in plastic cups, and warmed to 20 °C in a thermostatic chamber.

### 2.4. Determination of Moisture, Ashes, Sodium Chloride Content and Titratable Acidity

Moisture (method 925.15), ash (method 925.5), sodium chloride content (method 971.27), and titratable acidity (method 942.15) were determined by the procedures described by the Association of Official Analytical Chemists AOAC [19].

### 2.5. Vitamin C Content

Vitamin C content was determined according to procedure from the AOAC method 967.21 [19]. Briefly, 2 g of sample was mixed with 25 mL of filtered 3% metaphosphoric acid and 8% acetic acid solution in water. The amount of ascorbic acid in samples was calculated by titration with 4.9 mM 2,6-dichlorophenol-indophenol diluted in boiled water until pink color persisted for more than 30 s. A solution of 1 mg mL^−1^ ascorbic acid was also prepared and used as standard.

### 2.6. Evaluation of Color

Visual color of samples was quantified by using a CR-400 chroma-meter (Minolta CR-400, Konica Minolta, Tokyo, Japan) with CIE standard illuminant D65 and 0° viewing angle geometry. The instrument was set to the CIE L*a*b* color space and calibrated to a standard with calibration plate CR-A43 (Konica Minolta) before measurements were performed. For each sample, 5 readings were done, and color parameters were interpreted as follows:

L* = brightness (0%: black-colored objects; 100%: white-colored objects).

a* = negative values for green and positive values for red.

b* = negative values for blue and positive values for yellow.

Furthermore, relative saturation of the color (chroma, C) and hue angle (*h*) were calculated as follows:

(1) $$C={\left(a^{*2}+2b^{*2}\right)}^{0.5}$$

(2) $$h=\arctan\frac{b^{*}}{a^{*}}$$

The total change in color, ∆E*, was calculated according to the following formula:

∆E* = (∆L*^2^+ ∆a*^2^ + ∆b*^2^)^0.5^

where ∆L, ∆a*, ∆b* were the differences between the coordinate values of the homemade sample and the Pasteurized y raw, respectively [20].

### 2.7. Methanol and Acetone Extracts

Homogenized *salmorejo* samples were freeze dried at 4-13 Pa for 24 h using a Thermo Savant ModulyoD Freeze Dryer (Thermo Electron Corporation, Vantaa, Findland). To prepare extracts, 500 mg of freeze-dried sample was mixed with 5 mL of solvent (methanol/water (70:30, *v/v*) or acetone/water (80:20, *v/v*)) and shaken at RT on an orbital shaker for 3 h. Extracts were centrifuged at 2700× *g* for 15 min in a Selecta P Centromix centrifuge (Grupo Selecta, Barcelona, Spain), and the supernatants recovered. Remaining pellets were re-extracted with 5 mL of fresh solvent for a second time. Supernatants from both extractions were pooled and kept at 4 °C in the dark until analyzed the following day.

### 2.8. DPPH• Radical Scavenging Capacity

Scavenging activity of methanol and acetone extracts was determined according to the procedure described by Skrede et al. [21]. Homogenized *salmorejo* samples were freeze dried at 4–13 Pa for 24 h using a Thermo Savant ModulyoD Freeze Dryer (Thermo Electron Corporation, Vantaa, Findland). To prepare extracts, 500 mg of freeze-dried sample was mixed with 5 mL of solvent (methanol/water (70:30, *v/v*) or acetone/water (80:20, *v/v*)) and shaken at RT on an orbital shaker for 3 h. Extracts were centrifuged at 2700× *g* for 15 min in a Selecta P Centromix centrifuge (Grupo Selecta), and the supernatants recovered. Remaining pellets were re-extracted with 5 mL of fresh solvent for a second time. Supernatants from both extractions were pooled and kept at 4 °C in the dark until analyzed. Finally, 100 μL of methanolic or acetonic extract was mixed with 3.9 mL of 0.07 mM 1,1-diphenyl-1,1-picrylhydrazyl (DPPH) (Sigma-Aldrich, St. Louis, MO, USA). After thoroughly mixing, samples were incubated for 2 h at RT in the dark, and absorbance was measured at 515 nm in a spectrophotometer (UV-1700 Pharmaspec, Shimadzu, PRC) using ethanol as blank. Simultaneously, a calibration curve containing different concentrations (0–0.9 mM) of 6-hydroxy-2,5,7,8-tetramethylchroman-2-carboxylic acid (Trolox) (Sigma-Aldrich) was prepared. Absorbance values were expressed as mg Trolox Equivalents (TE) per g and DPPH• scavenging capacity (%).

### 2.9. Total Phenolic Content

Total phenols from *salmorejo* extracts were determined according to the procedure described by Muniandy et al. [22]. To that end, 100 μL of methanolic or acetonic extract were mixed with 7.9 mL of distilled water and 0.5 mL of 2 M Folin-Ciocalteu reagent. Mixture was incubated at RT for 5 min and then 1.5 mL of 20% (p/v) sodium carbonate solution was added. After incubating tubes for 2 h at RT in the dark, absorbance was measured at 765 nm using distilled water as a blank (Wasserlab Ecomatic, Navarra, Spain). Total phenolic content was determined using a gallic acid standard curve with concentrations ranging 400 to 0 μg mL^−1^, and results were expressed as mg gallic acid equivalent per milliliter (mg GAE mL^−1^).

### 2.10. Determination of Lycopene and β-Carotene Content

Lycopene and β-carotene content in *salmorejo* samples were determined following the procedure described by Darsan et al. [23]. To that end, 100 mg of freeze dried *salmorejo* powder was mixed thoroughly with 8 mL of a solvent consisting of a mixture of hexane, ethanol and acetone (2:1:1). Tubes were incubated for 30 min in the dark prior to adding 1 mL of deionized water and incubated again for 10 min to allow phases to separate. Blank samples were also prepared by adding 100 µL of water instead of sample. Lycopene and β-carotene content were determined by measuring absorbance of the upper layers of the resultant mixture at 503 nm and 478 nm, and applying the following formulas:

(3) $$\mathrm{Lycopene}\;\left(\frac{\mathrm{mg}}{\mathrm{kg}}\mathrm{fresh}\;\mathrm{wt}\right)=\frac{A_{503}\cdot 537\cdot 8\cdot 0.55}{0.1\cdot 172}$$

(4) $$\beta -\mathrm{Carotene}\;\left(\frac{\mathrm{mg}}{\mathrm{kg}}\mathrm{fresh}\;\mathrm{wt}\right)=\frac{[A_{478}-(A_{503}\cdot \;0.9285)]\cdot 533.85\cdot 8\cdot 0.55}{0.1\cdot 139}$$

where 537 g mol^−1^ and 533.85 g mol^−1^ are the molecular weights for lycopene and β-carotene, respectively. 8 mL is the employed volume of solvent, 0.55 is the volume ratio of the upper layer to the mixed solvents, 0.1 g is the weight of sample, and 172 mM^−1^ and 139 mM^−1^ are the extinction coefficients in hexane for lycopene and β-carotene, respectively.

### 2.11. Statistical Analysis

Quantitative variables were expressed as mean values ± SD. Statistical analyses were performed using R version 4.0.3 software (R Foundation for Statistical Computing, Vienna, Austria) on the RStudio platform. Differences among treatments were determined by using the Kruskal–Wallis test. Dunn test for multiple comparisons using rank sums was performed to determine which treatments differed from each other using “dunn.test” package. Samples were analyzed in triplicate, unless stated otherwise. *p*-values of less than 0.05 were regarded as statistically significant.

## 3. Results and Discussion

### 3.1. Physicochemical Properties

Table 1 shows the results of physicochemical properties of analyzed *salmorejo* samples. As can be observed, pasteurized samples showed the highest moisture content. In this sense, we must note that three out of five pasteurized samples analyzed and four out of seven raw samples analyzed declared water as an ingredient, whereas in homemade samples no extra water was added. Nevertheless, percentage of water added was not available in the ingredient list of commercial samples. Delgado-Andrade et al. [24] reported moisture content values of raw *salmorejo* of 81.69 ± 1.38%, which are similar to those described in this work. However, authors did not describe the percentage of ingredients employed in the recipe, and they also considered accompaniments (canned tuna and boiled egg) when determining moisture content values.

Regarding ash content, there were significant differences between pasteurized and raw samples, with homemade ones showing intermediate values. Observed results might be due to salt addition (an average of 7.52 ± 1.20 mg g^−1^ in pasteurized commercial products and 8.49 ± 2.51 mg g^−1^ in raw ones), that helps to prevent food spoilage caused by microorganisms, especially in raw *salmorejo*, and to the higher water content of pasteurized samples. There were not statistically significant differences between samples in terms of sodium chloride content. However, we must highlight that, although for this work the amount of salt added in homemade *salmorejo* was that recommended by the standardized recipe, in different households it can vary depending on personal preferences or awareness of salt intake.

There were not significant differences in titratable acidity nor in vitamin C content of analyzed *salmorejo* samples. Some studies have reported that pasteurization affects citric acid content of grapefruit juice, showing a reduction of the acid [25]. Nevertheless, according to our results, heat treatment did not affect acid content of samples. After determining tomato organic acids by HPLC, Verde-Mendez el al. (2011) found that citric and glutamic acid were presented at the highest mean concentration. However, the ingredients employed in the preparation of the dish, the state of ripeness of the plant, and genetic and environmental factors might affect acid content of the vegetables [26]. Furthermore, it should be noted that all but one commercial sample declared to contain vinegar (in a proportion that was not stated in the label), which also might contribute to the acidity of the product. Homemade *salmorejo* was prepared without adding vinegar, which can explain the smaller value found for titratable acidity. When it comes to vitamin C, some authors have described a reduction in vitamin C content in tomato puree when subjected to high pressure treatment or pasteurization [27], whereas others declared no differences after gazpacho (a traditional Spanish cold soup made of raw vegetables) samples underwent heat treatment [28]. Packaging also plays an important role, as losses in vitamin C are prevented when oxygen and light permeability are hampered, as happens when using Tetra Brik^®^ system [26,29], thus avoiding enzymatic oxidation of ascorbic acid to dehydroascorbic acid. In this sense, it should be noted that all pasteurized samples analyzed were packaged using Tetra Brik^®^ system whereas fresh ones did not. Regarding a_w_ values, although there were statistically significant differences between commercial and homemade samples, they were not meaningful from a technological perspective, as all average values were around 0.99, revealing that *salmorejo* would be a product highly susceptible to microbial spoilage were it not for its low pH value.

Finally, there were significant differences in pH values between raw (including homemade) and pasteurized samples. Pinilla et al. [28] reported that pH values of commercial gazpacho were not affected by heat treatment, so observed differences might be attributed to ripeness of tomatoes or genetic differences linked to the variety of tomato employed in the recipe. Besides, pH values could also depend on other factors, such as the addition of different amounts of vinegar [26,28,30].

### 3.2. Color Measurements

Objective color values of *salmorejo* samples are shown in Table 2. Raw and homemade samples showed significantly higher lightness (L*) and chroma (C), and were redder than pasteurized ones, whereas hue (H) values were higher for pasteurized samples. During *salmorejo* preparation, olive oil must emulsify with tomato puree, which entails a change in color from red to orange. The size of oil droplets and their concentration influence the physical-chemical and sensory properties of the product. The larger the size of oil droplets, the lesser the L* values and the higher the color of the emulsion. Similarly, emulsion brightness increases as concentration of oil droplets do [31]. This might explain the observed results, as raw *salmorejo* samples declared in the label higher oil content than pasteurized ones. On the other hand, color values are affected by heat treatment. In this sense, Sánchez-Moreno et al. [27] reported losses in color in tomato puree samples after heating them at 70 °C for 30 s and at 90 °C for 1 min. Similarly, Shi et al. [32] described a reduction in color in tomato juice that was accelerated by high temperatures and long treatments, mainly due to the degradation of lycopene. Nevertheless, Mirondo et al. [33] reported that heat treatment did not have any effect on hue angle, L* and a* values in tomato juices and sauces. In this work, however, an increase in the values of hue angle, which is associated with an increase in the degree of browning [34], as well as a reduction in a* values, which might be associated with lycopene degradation, have been observed in pasteurized samples. Color differences (ΔE) calculated from the recorded values were 6.30 and 1.48 for pasteurized and raw samples, respectively. It is assumed that an inexperienced observer might be able to differentiate two products when color difference is greater than 3.5. Thus, it could be stated that pasteurization treatment modifies the color of *salmorejo* in a way that it is perceptible to human eye [20].

### 3.3. DPPH• Radical Scavenging Capacity

Antioxidant activities of *salmorejo* extracts are presented in Table 3. It is noteworthy to mention that results differed depending on the solvent employed to perform the extraction. In this sense, when results were expressed per dry weight, it was observed that methanol extracts from pasteurized samples inhibited DPPH• radical to a larger extent than the ones from homemade samples, being raw samples’ results somewhere in between. However, this effect was not observed after analyzing acetone extracts. Differences between solvents might be due to different antioxidant molecules being extracted in the process [35]. It has been described that thermal treatment might deactivate oxidative enzymes, preventing loss of antioxidant compounds, and the appearance of Maillard reaction products might also contribute to inhibit oxidative enzymes that cause enzymatic browning [36]. On the other hand, a cultivar-dependent decrease in antioxidant compounds has been observed after subjecting EVOO to heating, with tocopherols and polyphenols being largely affected. However, oleic acids, sterols, and squalene might be more stable against oxidation [37]. When expressing DPPH• values considering the water content of samples, it was observed that homemade *salmorejo* provided a higher content of antioxidant molecules when analyzing acetone extracts.

### 3.4. Total Phenolic Content

The total phenolic content (TPC) of *salmorejo* samples analyzed is summarized in Table 4. Results showed that there were not significant differences between TPC of raw and pasteurized freeze-dried samples. However, when TPC values were expressed considering moisture content of samples, statistically significant differences were found between raw and homemade samples on the one hand and pasteurized products on the other. These findings would imply that homemade and raw *salmorejo* samples are richer in polyphenols than pasteurized samples.

According to the standardized recipe of *salmorejo*, tomato makes up approximately 75% of the product, bread accounts for slightly less than 15%, and olive oil amounts to around 8%. TPC values for raw tomato published in Phenol-Explorer database are in the range of 0.45 ± 0.16 mg g^−1^ FW [38]. Thus, considering the abundance of tomato in *salmorejo*, which is the main source of phenolic compounds in the product, TPC values obtained in the present research work are within the range of expected results. Although “plum tomato” is the recommended variety to prepare the recipe, different tomato cultivars are frequently employed, which could alter the quantity of phenolic compounds provided by *salmorejo*, as phenolic content of tomatoes is influenced by genotype and agronomic, geographical and seasonal factors, by the degree of ripeness in fruits, by solar UV radiation available, and by whether the skin and seeds of fruits are removed during the processing [39,40,41]. Content in phenolic compounds is also influenced by the type of olive oil used in the preparation. In this sense, the use of EVOO (the one thoroughly recommended in the recipe) would increase the provision of phenols in comparison to refined olive oil or different vegetable oils [42]. Virgin olive oil (VOO) is obtained from olives only by mechanical-physical processes, and without additional refining. When VOO has no defects from an organoleptic point of view, and its acidity level do not exceed 0.8%, it is considered EVOO. EVOO has the highest content in polyphenols. On the other hand, phenolic compounds and to lesser degree squalene are lost during refining process [38,43]. The content of phenolic compounds in other different cold-pressed plant oils can be found in the work of Siger et al. (2008) [43]. Furthermore, it should be noted that the addition of oil to tomato matrix appears to enhance the extraction of phenolic compounds, leading to higher phenolic content in tomato sauces [44,45]. Regarding commercial products, differences in the proportion of ingredients used in the recipe might explain the heterogeneity of results. Unfortunately, the percentage of ingredients was not always declared on the label, which together with the addition of non-traditional ingredients, such as pepper, onion, etc., made it difficult to ascribe observed differences in TPC values to a single variable.

When it comes to the influence of thermal treatment over TPC, Perez-Conesa et al. [46] described a reduction in TPC of tomato puree when food products underwent pasteurization at 98 °C for 40 s. Comparable results were obtained by Pinilla et al. [28], who found higher TPC in slight pasteurized (70 °C, 30 s) gazpacho samples than in traditional pasteurized (92 to 95 °C, 1 min) gazpacho samples. Vallverdú-Queralt et al. [18], observed a general reduction of phenolics in tomato sauces during cooking process, except for caffeic acid and tyrosol. However, Gahler et al. [47] reported an increase in TPC in tomato products after heat treatments, and Dewanto et al. [48] showed that thermal processing at 88 °C for 2, 15, and 30 min did not produce significant changes in TPC of tomato. Martínez-Huélamo et al. [45] have reported an increase in the bioaccessibility, extractability and bioavailability of phenolics from tomato after mechanical and thermal treatments, probably due to the release of phenolics from the matrix through breakdown of cellular constituents and the inactivation of oxidative and hydrolytic enzymes released by cell disruption.

### 3.5. Lycopene and β-Carotene Content

Lycopene and β-carotene content of *salmorejo* samples is presented in Table 5. Obtained results indicated that raw samples had a larger lycopene content that pasteurized samples. Loses in lycopene content have been reported when tomato and oil mixtures are heated at temperatures above 100 °C. The presence of oil, which solubilizes crystalline lycopene, makes it more susceptible to thermal degradation. Furthermore, intense mechanical treatment of tomatoes makes lycopene less protected by the food matrix, and thus more sensible to isomerization [49]. Lycopene content of *salmorejo* is also influenced by manufacturing process: tomato peel contains more lycopene than tomato pulp. Therefore, if peel is not removed, the final lycopene content of *salmorejo* would be higher [50]. It should also be noted that lycopene content is influenced by tomato variety, state of ripeness of the fruit, and date of harvesting [51].

On the other hand, according to results shown in Table 5, β-carotene content of *salmorejo* samples was not influenced by heat treatment. However, when the total amount of water of the product was considered, raw *salmorejo* samples would supply a larger amount of β-carotene. β-Carotene, the second main carotenoid in tomato after lycopene, seems to be stable during the thermal treatment of tomato paste [52]. Georgé et al. [53] did not find processing to affect carotenoid content in red tomatoes. Similarly, Perez-Conesa et al. [46] found that a vapor stream at 82 °C for 2 min of tomato puree did not affect the content of lycopene or β-carotene, and Abdul-Hammed et al. [54] agreed that lycopene and β-carotene are better retained when heat-treating tomato paste at low-temperature and short-time, reporting a severe reduction of both components after heating tomato paste at 120 °C for 90 min.

## 4. Conclusions

This study has strengths and limitations. Among the latter, it should be mentioned that to properly compare the effects of heat treatment on physicochemical and functional properties of *salmorejo*, home-made samples should have undergone different treatment conditions to produce equivalent time-temperature profiles to mimic those performed in the industry, as for example described in Anese et al. (2002) [55]. Secondly, in depth analysis of methanolic and acetonic extracts would be necessary to offer a complete explanation which join all the results showed in our study.

In conclusion, this work provides a deeper understanding on the physical-chemical and functional properties of commercial raw and pasteurized *salmorejo* that can be purchased in Spanish supermarkets and compares them with homemade *salmorejo* samples. Pasteurized samples analyzed showed differences that could be ascribed to an extra addition of water in the recipe. Furthermore, main differences attributed to heat treatment entailed changes in color parameters and losses in lycopene content. From the food industry standpoint, color is a key attribute that determines consumers acceptance of a product, and potential buyers might reject goods that no longer preserve qualities associated with homemade ones. Other differences found between samples could be due to the addition of ingredients such as vinegar, which might affect pH values. Obtained data also suggest that the use of EVOO resulted in a higher supply of antioxidant molecules, as can be assessed when comparing commercial samples to homemade ones, thus the importance of checking the list of ingredients to ascertain the percentage as well as the type of oil employed in the recipe prior to purchasing the product. Further studies are needed to separate the effects of thermal treatment from those of the ingredients themselves.

## Figures and Tables

**Table 1:** Physical-chemical parameters of *salmorejo* samples (mean ± SD).

Sample	Moisture Content (%)	Ashes (%)	Sodium Chloride (mg g^−1^)	Titratable Acidity (%)	Vitamin C (μg g^−1^)	a_w_	pH
Pasteurized	85.80 ± 1.22 ^a^	1.09 ± 0.11 ^a^	7.52 ± 1.20 ^a^	0.44 ± 0.04 ^a^	79.01 ± 30.85 ^a^	0.990 ± 0.016 ^a^	3.91 ± 0.06 ^a^
Raw	81.07 ± 6.01 ^b^	1.29 ± 0.24 ^b^	8.49 ± 2.51 ^a^	0.46 ± 0.13 ^a^	68.12 ± 48.05 ^a^	0.993 ± 0.003 ^a^	4.41 ± 0.36 ^b^
Homemade	74.41 ± 0.06 ^b^	1.11 ± 0.23 ^ab^	8.95 ± 0.21 ^a^	0.36 ± 0.10 ^a^	74.57 ± 9.38 ^a^	0.989 ± 0.001 ^b^	4.23 ± 0.26 ^b^
*p*-value	0.014	0.030	0.4907	0.4566	0.2468	0.010	<0.001

Kruskal-Wallis (type of treatment). SD, standard deviation. Means with superscripts having different letters are significantly different (Dunn’s test).

**Table 2:** CIE L*a*b*, chroma (C) and hue (H) Color parameters of *salmorejo* samples (mean ± SD).

Sample	L*	a*	b*	C	H
Pasteurized	42.43 ± 2.70 ^a^	11.97 ± 2.48 ^a^	26.48 ± 4.02 ^a^	29.10 ± 4.31 ^a^	1.15± 0.07 ^a^
Raw	44.65 ± 2.22 ^b^	17.73 ± 1.62 ^b^	26.08 ± 5.10 ^a^	31.76 ± 3.74 ^b^	0.96 ± 0.13 ^b^
Homemade	46.12 ± 1.21 ^b^	17.07 ± 3.63 ^b^	26.77 ± 1.36 ^a^	31.85 ± 2.78 ^ab^	1.01 ± 0.09 ^b^
*p*-value	0.0004	<0.001	0.5602	0.0409	<0.001

Kruskal-Wallis (type of treatment). SD, standard deviation. Means with superscripts having different letters are significantly different (Dunn’s test).

**Table 3:** Radical scavenging activity (expressed in mg Trolox equivalent (TE) per g freeze dried sample or per g fresh weight) of *salmorejo* samples (mean ± SD).

	DPPH (mg Trolox g^−1^ DW) (% Inhibition)		DPPH (mg Trolox g^−1^ FW) (% Inhibition)	
Sample	Methanol	Acetone	Methanol	Acetone
Pasteurized	0.88 ± 0.23 ^a^	1.01 ± 0.20 ^a^	0.13 ± 0.02 ^a^	0.15 ± 0.02 ^a^
	(15.88 ± 4.20)	(18.31 ± 3.69)	(2.35 ± 0.38)	(2.75 ± 0.38)
Raw	0.68 ± 0.31 ^ab^	0.82 ± 0.33 ^a^	0.11 ± 0.03 ^a^	0.14 ± 0.04 ^a^
	(12.22 ± 5.57)	(14.86 ± 5.89)	(2.04 ± 0.59)	(2.54 ± 0.71)
Homemade	0.46 ± 0.06 ^b^	0.78 ± 0.11 ^a^	0.12 ± 0.01 ^a^	0.20 ± 0.03 ^b^
	(8.25 ± 1.04)	(14.19 ± 2.03)	(2.11 ± 0.26)	(3.63 ± 0.52)
*p*-value	0.0377	0.1423	0.2976	0.0057

Kruskal-Wallis (type of treatment). SD, standard deviation. Means with superscripts having different letters are significantly different (Dunn’s test). DW: dry weight; FW: fresh weight.

**Table 4:** Average content of total polyphenols (mg gallic acid equivalent (GAE) per g freeze dried sample or per g fresh weight) of *salmorejo* samples (mean ± SD).

	TPC (mg GAE g^−1^ DW)		TPC (mg GAE g^−1^ FW)	
Sample	Methanol	Acetone	Methanol	Acetone
Pasteurized	1.83 ± 0.44 ^a^	1.59 ± 0.33 ^a^	0.27 ± 0.04 ^a^	0.24 ± 0.03 ^a^
Raw	1.89 ± 0.60 ^a^	1.77 ± 0.43 ^a^	0.31 ± 0.06 ^b^	0.30 ± 0.05 ^b^
Homemade	1.42 ± 0.16 ^a^	1.34 ± 0.06 ^a^	0.36 ± 0.04 ^b^	0.34 ± 0.02 ^b^
*p*-value	0.5037	0.1309	0.0048	0.0026

Kruskal-Wallis (type of treatment). SD, standard deviation. Means with superscripts having different letters are significantly different (Dunn’s test). DW: dry weight; FW: fresh weight.

**Table 5:** Lycopene and β-carotene content (expressed in mg lycopene or β-carotene per g freeze-dried sample or per g fresh weight) of *salmorejo* samples (mean ± SD).

Sample	Lycopene (mg kg^−^^1^ DW)	Lycopene (mg kg^−^^1^ FW)	β-Carotene (mg kg^−^^1^ DW)	β-Carotene (mg kg^−^^1^ FW)
Pasteurized	47.82 ± 22.84 ^a^	6.67 ± 2.99 ^a^	22.06 ±5.17 ^a^	3.10 ± 0.61 ^a^
Raw	165.80 ± 87.60 ^b^	28.07 ± 8.06 ^b^	26.84 ±6.79 ^a^	4.82 ± 1.03 ^b^
Homemade	115.90 ± 69.39 ^ab^	29.64 ± 17.70 ^b^	22.98 ± 5.74 ^a^	5.88 ± 1.46 ^b^
*p*-value	0.0002	<0.001	0.1694	0.0013

Kruskal-Wallis (type of treatment). SD, standard deviation. Means with superscripts having different letters are significantly different (Dunn’s test). DW: dry weight; FW: fresh weight.
